# The preparation of polyvinyl imidazole-functionalized magnetic biochar decorated by silver nanoparticles as an efficient catalyst for the synthesis of spiro-2-Amino-4*H*-pyran compounds

**DOI:** 10.1038/s41598-022-25857-0

**Published:** 2022-12-24

**Authors:** Leila Mohammadi, Majid M. Heravi, Asma Saljooqi, Pourya Mohammadi

**Affiliations:** 1 Department of Chemistry, Faculty of Physics and Chemistry, Alzahra University, Vanak, PO. Box 1993891176, Tehran, Iran; 2 Department of Chemistry, Shahid Bahonar University of Kerman, Kerman, Iran

**Keywords:** Environmental chemistry, Natural hazards

## Abstract

The silver nanoparticle was synthesized by developing poly (1-vinylimidazole) on the surface of magnetized biochar (the stem and roots of *Spear Thistle*) (biochar/Fe_3_O_4_/PVIm/Ag). This nanocomposite was characterized by Fourier-transformed infrared spectroscopy (FTIR), powder X-ray diffraction (XRD), vibrating sample magnetometer (VSM), scanning electron microscopy-energy dispersive X-ray spectroscopy (SEM–EDS), and transmission electron microscopy (TEM). The SEM and TEM images of the nanocatalyst, biochar/Fe_3_O_4_/PVIm/Ag-NPs, confirmed the observation of microscopic sheets of biochar. The catalytic activity of these Ag NPs was tested via multicomponent reaction plus reusing to successful formation of 2-amino-4H-pyran and functionalized spirochromen derivatives. The prepared nanocatalyst was easily separated by an external magnet and reused in repeating coupling reaction cycles four times without remarkable activity loss. The catalyst showed great efficiency and reusability, thus making it an ideal candidate for catalytic purposes in several organic transformations.

## Introduction

Biochar can be used as a promising material to replace expensive carbon materials as support for reactant species. However, they suffer from two or three disadvantages, insufficient support dependencies, and extensive active sites^1,2^. Biochar can be dark igneous carbon obtained from thermal decomposition forms (such as direct pyrolysis, hydrocarbonation, and gasification) of specific carbon-rich biomass or explosion in an oxygen-limited environment^3^. The ever-expanding ecological concerns have generated many efforts on the improvement of desirable natural heterogeneous catalysts. One of the powerful methods for preparing biochar is the hydrocarbonation of biomass at relatively mild temperatures. Determined hydrothermal biochars are known as hydrochars and have attracted much attention due to their simple arrangement strategy. This magnificence of biomaterials may be significantly implemented with various equipment^4,5^.

Compared to solids and bulk compounds with high ratios of surface atoms with high energy, metal nanoparticles show unique but attractive chemical and physical properties^6,7^. Silver nanoparticles are one of the most used metal nanoparticles, and can catalyze some organic transformations. To produce silver nanoparticles, the salt is usually reduced using various reducing agents, such as hydrazine hydrate^8,9^. The most advantage is that an expansive amount of nanoparticle material can be produced. The precipitation strategy is probably the best and most useful chemical method to synthesize the desired nanoparticles^10^.

Hence, modification of biochar, especially with Fe_3_O_4_ nanoparticles, may advance the potential recovery of biochar in absorbent media. Biochar^11^, multilayer carbon nanotubes^12^, graphene^13^, clay^14^, engineered carbon, carbon microspheres^15^, in terms of small size, hydrophilic groups used on their surface can be attention. One of the best types of supports is polymers, which are widely used to support various types of catalysts^16,17^. Polyimidazole (PVIm) gives more compounds than carbon materials because the connection of nitrogen atoms in the carbon structure can have excellent adaptability and simplicity in the arrangement and reconstruction of its chemical, electrical and optical properties^18^. In addition, functionalities can serve as the basis for equilibrating catalytic species through non-covalent interactions. PVIm has been used in particular as a support to immobilize silver nanoparticles to produce Ag/PVIm that may catalyze multi-component reactions^18^.

Currently, multicomponent reactions (MCRs) have attracted much attention in both academia and industry due to their specific combinatorial effectiveness, intrinsic atom economy, and stable integration^19^. Many diverse and exciting heterocyclic structures, especially those with synthetic appeal as adjuncts capable of safe biological detection, were generated through MCRs^20,21^.

MCRs are convenient for the sensible set up of chemical libraries of essentially associated, medicinally primary pharmaceutical analog compounds^22^. Hence, the arrangement of recent multi-component reactions has attracted considerable attention, especially in the area of mild detection and integration of complex particles and characterized compounds. MCRs, especially those used in aqueous environments, have received tremendous attention in engineered natural chemistry these days for the planning of important chemical and natural compounds through the use of concerted methods, atom economy, and green methods^23,24^. Subsequently, in general, old pot design MCRs reduce chemical waste, which can have a large effect in shortening the reaction times of starting materials and providing high yields of common chemicals^25^ Although possible, the loading of spirodioxin compounds for the common synthetic drug use of alkaloids, the diverse effects of alkaloids as scientific pharmaceutical intermediates, and the very obvious biological effects are a very long purpose^26^.

**Figure 1:**
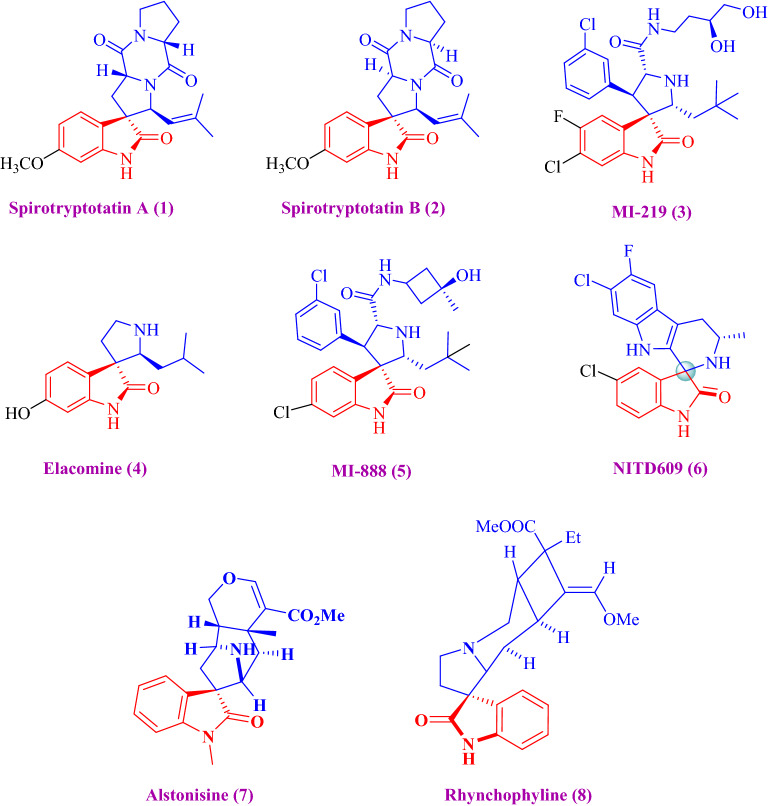
Spirooxindoles compound exhibiting biological enterprises.

As shown in Fig. 1, the reasons for the reduced efficiency of the three 3-spiroisin cores have attracted the interest of synthetic organic chemists in the synthesis of these adducts.^27^. That is why there are some reports^28^ of different catalysts such as l-proline^29^, TEBA^30^, and NH_4_Cl in MCR for the synthesis of leucine derivatives in an aqueous medium. Their use has increased the difficulty of purification used in these reactions.

Heterocyclic compounds^31,32^ synthesized through MCR^33^ in the presence of heterogeneous catalysts in an aqueous medium are of particular importance^34,35^.

**Scheme 1:**
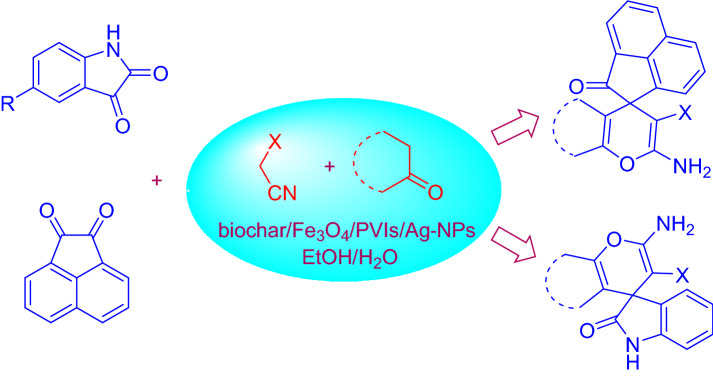
A potent one-pot blend of rationalized spirochromenes in terms of biochar/Fe_3_O_4_/PVIm/Ag catalyst.

A silver nanoparticle was synthesized by developing poly(1‑vinylimidazole) on the surface of magnetic biochar (biochar/Fe3O4/PVIm/Ag). The catalytic activity of the heterogeneous catalyst was investigated for the synthesis of spiro-2-Amino-4H-pyrans (spirochromenes) through multi-component reactions. The reusing test confirmed that the catalyst had relative stability and reusability, making it a good candidate for catalytic purposes. The catalyst can be reused several times in repeating the coupling reaction cycles with some loss of its activity. It should be noted that MCRs have attracted much attention in both the scientific and industrial worlds due to their specific engineering viability, intrinsic particle economy, and achievable integration^42–44^ (Scheme 1).

## Experimental

### Materials

All chemical substances have been used as bought from Sigma-Aldrich and Merck Companies. Tetraethyl orthosilicate (tetraethoxysilane) (98% w/w), ethanol (99.5% w/w), (3-aminopropyl) triethoxysilane (95% w/w), HNO_3_ (65% w/w), and silver nitrate (≥ 99.0%), potassium persulphate (99.99%), hydrazine hydrate (24–26% in H_2_O (RT)), isatin (97%), acenaphthene quinone (97%), malononitrile (98%), ethyl cyanoacetate (98%), dimedone (95%), barbituric acid ethyl acetoacetate (99%), 4-hydroxycoumarin (98%), three-methyl-1H-pyrazole-five (4H)-one (98%), α-naphthol (99%), β-naphthol (99%), n-hexane (99%), ethyl acetic acid (99%), and desired derivations have been provided from the Sigma-Aldrich Company. The stem and roots of the *Spear Thistle* were purchased from a local shop in Tehran (in Iran). The leaves were purchased from a local shop in Tehran. The plant we used in this work is a plant that is found in abundance in local shops and is not wild and endangered. This study complies with relevant institutional, national, and international guidelines and legislation.

### Biomass material

**Figure 2:**
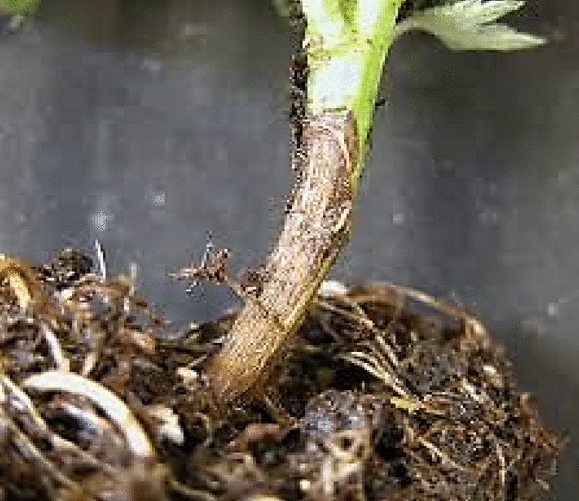
The stem and roots of Spear Thistle-derived.

The raw biomass material in this paper was the stem and roots of *Spear Thistle* (Fig. 2). Firstly, the material was washed several times using deionized water to remove the impurities. Then, the stem and roots of *Spear Thistle* were crushed within a particle size range of 0.9–2 mm. After that, the samples were poured into a Teflon-lined hydrothermal autoclave reactor, then the deionized water (70 mL) was added to it and put in an oven by adjusting the temperature at 185 °C for 24 h. Finally, the product was centrifuged (6000 rpm) for 15 min, washed several times with deionized, water and dried at room temperature.

### Synthesis of Fe_3_O_4_/biochar

The synthesis of Fe_3_O_4_/biochar nanocomposites was conducted as follows: Generally, biochar (0.3 g) was dispersed in 120 mL of deionized water for 30 min. In the next stage, 1.37 g FeCl_3_·6H_2_O and 0.5 g FeCl_2_·4H_2_O were added to this mixture and well stirred at 60 °C for 6 h. After this time the ammonium solution (11 mL) was added dropping. Then the magnetic Fe_3_O_4_/biochar was prepared after 1 h at 60 °C. Finally, the magnetic biochar was cooled then separated via a neodymium, magnet and washed twice with deionized water.

### Synthesis of biochar/Fe_3_O_4_/PVIm

For this purpose, 0.6 g of magnetic biomass was stirred in ethanol (20 mL) for 1 h. Then 1-vinyl imidazole (10 mmol, 1 mL) was dissolved in ethanol (2–3 mL) to form a homogeneous solution then was added dropwise to magnetic biomass, and stirred for 2 h. Afterward, potassium persulphate (KPS) (0.06 g dissolved in 2–3 mL deionized water) was added dropwise to the above mixture as an initiator. Then, the mixture was stirred at 70–75 °C under an N_2_ atmosphere. Eventually, this product was washed with water and ethanol several times using an external magnetic field and dried at room temperature.

### Synthesis of biochar/Fe_3_O_4_/PVIm/Ag nanocatalyst

First, a certain amount of AgNO_3_ (0.025 g dissolved in 50 mL deionized water) was prepared and stirred as long as the solution was obtained clear. After that o.3 g of based catalyst biochar/Fe_3_O_4_/PVIm was added to the above solution and then stirred for 6 h at ambient temperature. Separately, 0.5 mL of hydrazine hydrate was dissolved in 5 mL of deionized water, next, 1.0 mL of this solution was added to biochar/Fe_3_O_4_/PVIm/Ag^+^ and stirred for 24 h. Eventually, the product was eluted with water and dried at room condition. A novel biochar/Fe_3_O_4_/PVIm/Ag nanocatalyst was obtained for the synthesis of spiro-2-Amino-4*H*-pyrans (spirochromenes) through multi-component reactions and reduction of nitro aryl aromatic compounds.

### Formation of spiro-2-amino-4*H*-pryans

A mixture of 1.0 mmol of isatin (or acenaphthenequinone), 1.0 mmol of malononitrile (or ethyl cyanoacetate), and 1.0 mmol of 1,3-diketones (dimedone, barbituric acid ethyl acetoacetate), (or 4-hydroxycoumarin/three-methyl-1*H*-pyrazole-five (4*H*)-one/α-naphthol or β-naphthol) was stirred with H_2_O/EtOH (5 mL) in the presence of biochar/Fe_3_O_4_/PVIm/Ag (0.03 g) as a heterogeneous catalyst under appropriate time reflux conditions which turned into proven in Tables 1 and 2.

**Table 1:** Investigating the effect of solvent on the synthesis of derivative 12a via MCR.

Entry	Solvent	Temperature	Catalyst amount (mol%)	Time (min)	Yield (%)
1	DMF	Reflux	0.025	160	70
2	CH_3_CN	Reflux	0.025	90	65
3	Toluene	Reflux	0.025	100	60
4	CH_2_Cl_2_	Reflux	0.025	167	45
5	H_2_O	Reflux	0.025	112	45
7	EtOH	Reflux	0.025	108	65
8	EtOH/H_2_O	Reflux	–	370	Trace
9	EtOH/H_2_O	Reflux	0.03	9	98
10	EtOH/H_2_O	r.t.	0.025	104	80
11	EtOH/H_2_O	Reflux	0.01	85	75
12	EtOH/H_2_O	Reflux	0.05	55	92
13	EtOH/H_2_O	Reflux	0.05	85	93

**Table 2:** Synthesis of spiro-2-amino-4*H*-pyrans (spirochromenes) (**12a-u**) within the presence of biochar/Fe_3_O4/PVIm/Ag using MCR.

Entry	R	Ar	X	Time (min)	Product	Yield (%)	m.p. (ºC) Obs	m.p. (ºC) Lit
1	H		CN	9	**12a**	98	286–288	286–287^36^
2	H		CN	10	**12b**	97	277–278	278–280^37^
3	H		CN	27	**12c**	91	291–293	292–294^38^
4	H		CN	12	**12d**	95	280–283	281–283^39^
5	H		CN	35	**12e**	87	254–256	255–256^40^
6	H		CN	17	**12f**	89	221–225	220–222^41^
7	H		CN	19	**12g**	85	235	233–235^42^
8	H		CO_2_Et	16	**12h**	78	259–261	260^43^
9	H		CO_2_Et	25	**12i**	92	268–270	269–271^44^
10	H		CO_2_Et	32	**12j**	79	251–254	251–253^28^
11	H		CO_2_Et	22	**12k**	85	284–286	285–287^45^
12	H		CO_2_Et	52	**12l**	76	173–176	176^43^
13	H		CO_2_Et	37	**12m**	82	229–231	2229^46^
14	Cl		CN	19	**12n**	88	287–290	289–290^47^
15	Cl		CN	18	**12o**	89	290–294	291–293^48^
16	Cl		CN	20	**12p**	87	> 300	> 300^49^
17	Cl		CN	18	**12q**	89	231–233	230–232^49^
18	Cl		CN	25	**12r**	87	262–265	263–265^50^
19	Cl		CN	25	**12s**	87	> 300	> 300^51^
20	Cl		CO_2_Et	35	**12t**	85	270–272	271–272^52^
21	Cl		CO_2_Et	32	**12u**	87	246–248	246–248^49^

This reaction was monitored using TLC (n-hexane/ethyl acetic acid, 3:2). After the completion of the reaction, it was cooled to room temperature, and the prepared nanocatalyst was isolated via an external magnet. The obtained product was dried and crystallized in hot ethanol.

In addition, to ensure the reproducibility of the catalyst in optimal conditions, the reaction was performed three times, and the nanocatalyst was tested. The yields presented are the average of three replicates.

## Results and discussion

### Characterization of catalyst

After the synthesis of the final nanocatalyst, it was characterized by several techniques including FTIR, FESEM, EDS-Mapping, XRD, TEM, VSM as well as ICP.

**Figure 3:**
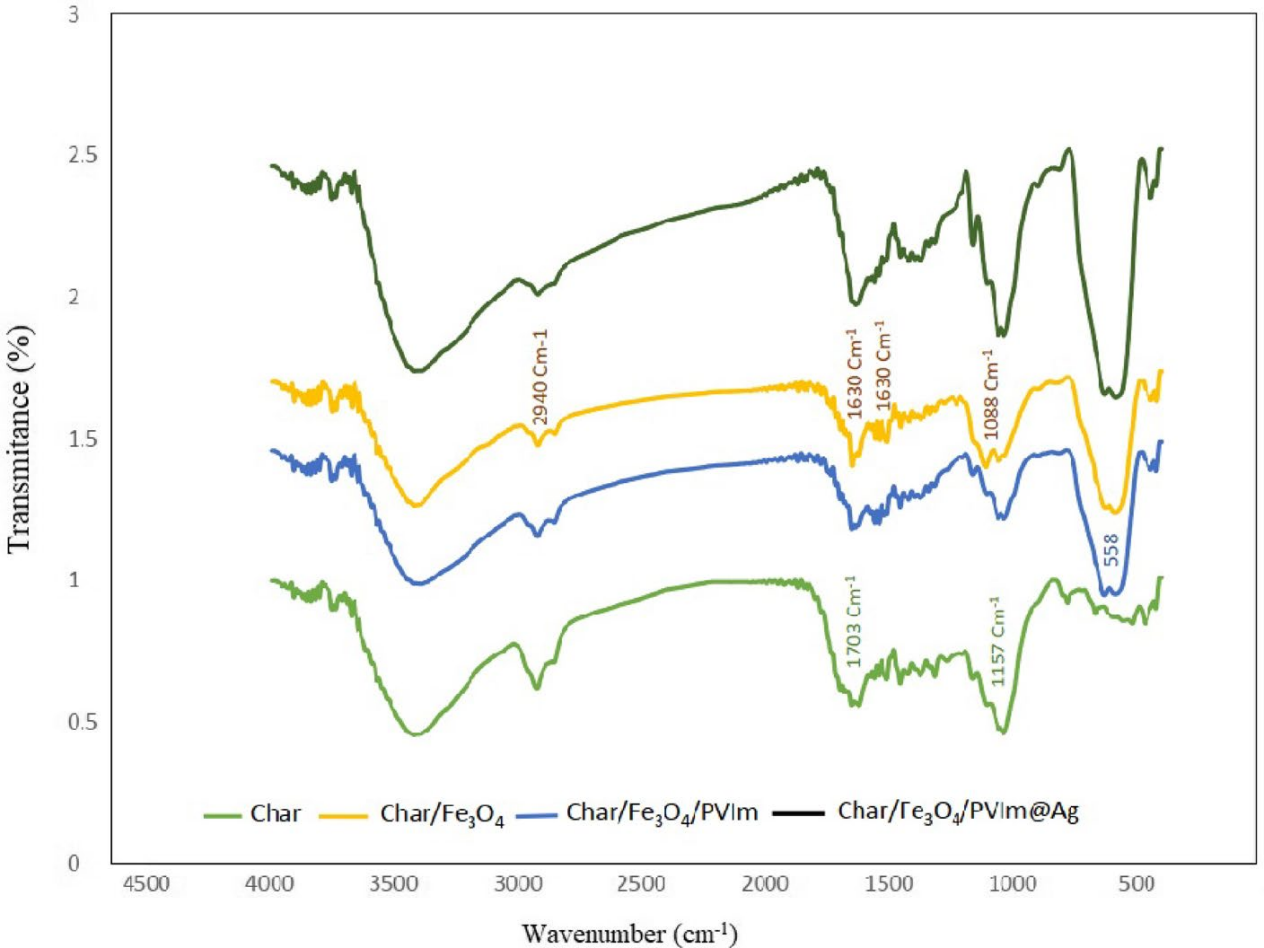
Fourier transform infrared (FT–IR) of biochar/Fe_3_O_4_/PVIm/Ag nanocatalyst.

FT-IR spectra were used to approve the existence of functional groups in biochar, biochar/Fe_3_O_4_, biochar/Fe_3_O_4_/PVIm, and biochar/Fe_3_O_4_/PVIm/Ag compounds (Fig. 3).

The spectrum of biochar/Fe_3_O_4_ is in great agreement with the literature, showing approximately 3388 cm^−1^ (–OH), 2927 cm^−1^ (–CH), and 1703 cm^−1^ (–C=O) characteristic groups. It is speculated that the 1627 cm^−1^ (–C=C) and 1156 cm^−1^ (–CO) in biochar contained distinct functions in their structure. In addition, the peak of biochar/Fe_3_O_4_ attended at 450–560 cm^−1^ can be related to Fe–O stretch vibration. Although the characteristic peaks of PVIm and Ag NP can be identified in the FTIR spectra of the cast, the spectra of these components overlap with functional groups of biochar and Fe_3_O_4_, the FTIR spectrophotometer cannot confirm the forming of biochar/Fe_3_O_4_/PVIm/Ag-NP.

**Figure 4:**
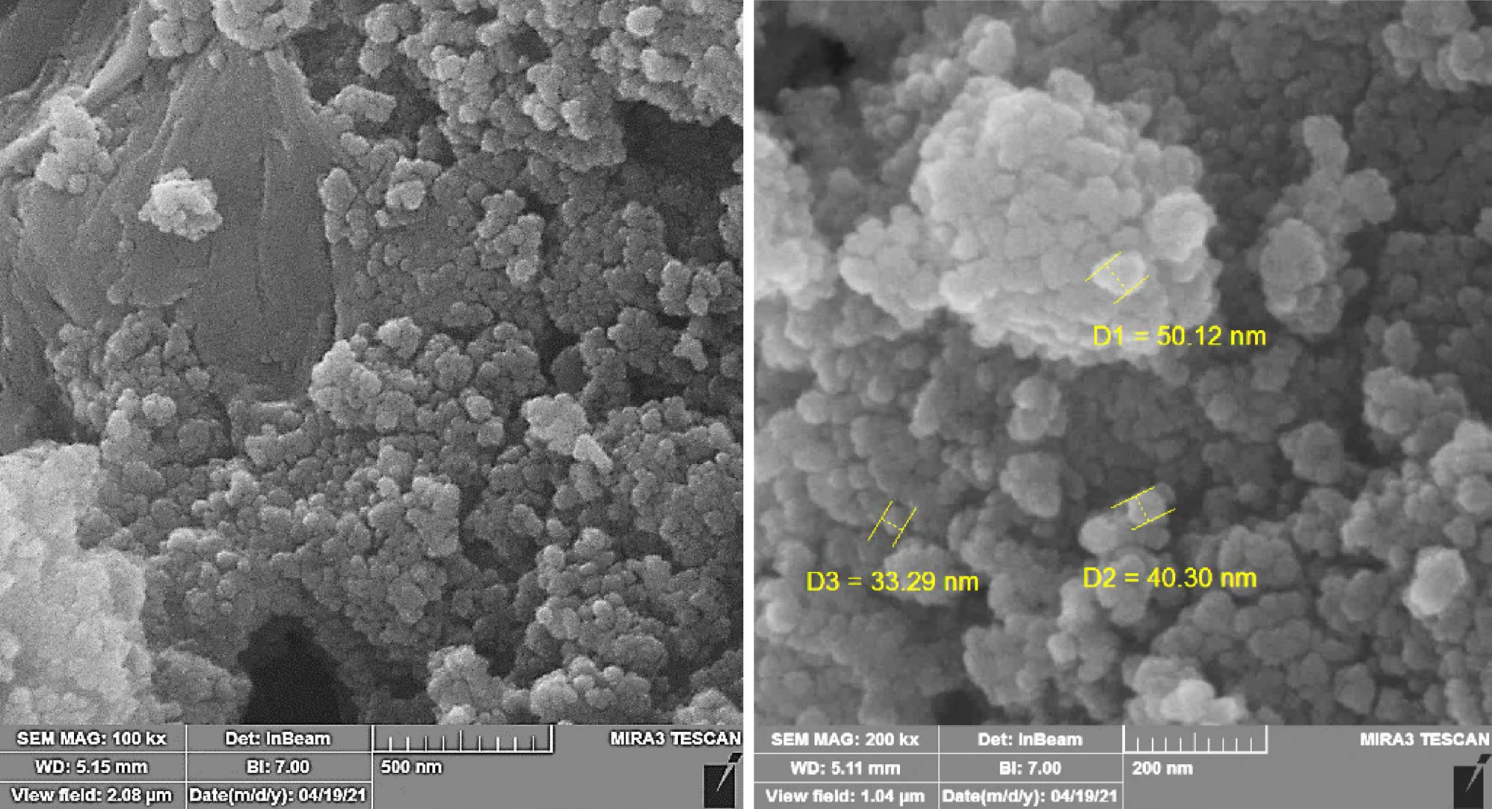
FE–SEM image of biochar/Fe_3_O_4_/PVIm/Ag nanocatalyst.

Dispersion and morphology of the biochar/Fe_3_O_4_/PVIm/Ag particles were investigated via field emission-scanning microscopy (FE–SEM) as seen in Fig. 4. The morphology of biochar/Fe_3_O_4_/PVIm/Ag was seen in FE–SEM images with small particles of Fe_3_O_4_ and Ag with almost spherical morphology, which can be recognized are stuffed together and dispersed on the biochar surface. Additionally, the size of synthesized particles is 33.29 nm to 50.12 nm, which recommends they're nanosized.

**Figure 5:**
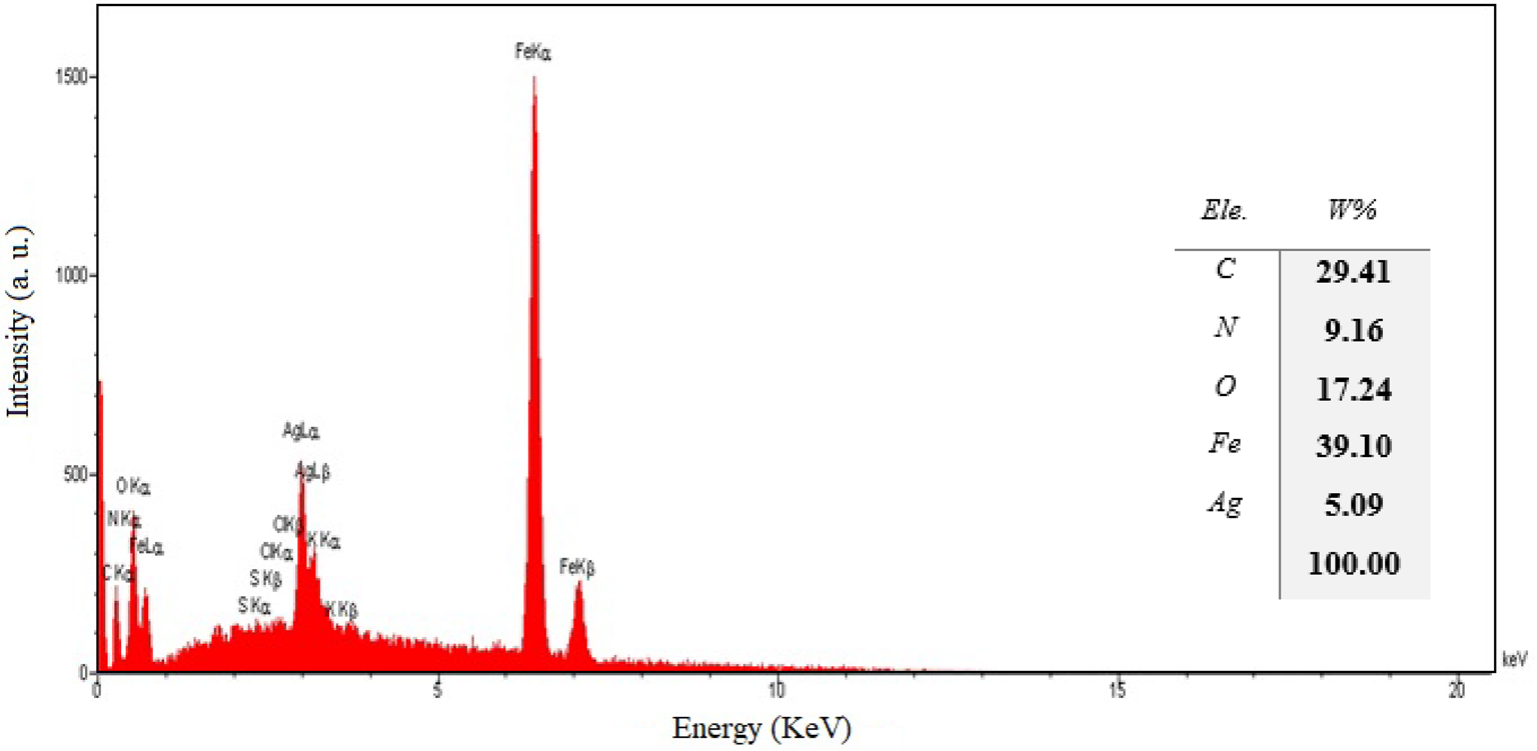
EDS of the biochar/Fe_3_O_4_/PVIm/Ag nanocatalyst.

The EDX examination of the nanocatalyst, as appeared in Fig. 5, further illustrates the presence of Ag, Fe, C, O, and N elements as well as confirms the successful immobilization of Ag nanoparticles in biochar/Fe_3_O_4_/PVIm.

**Figure 6:**
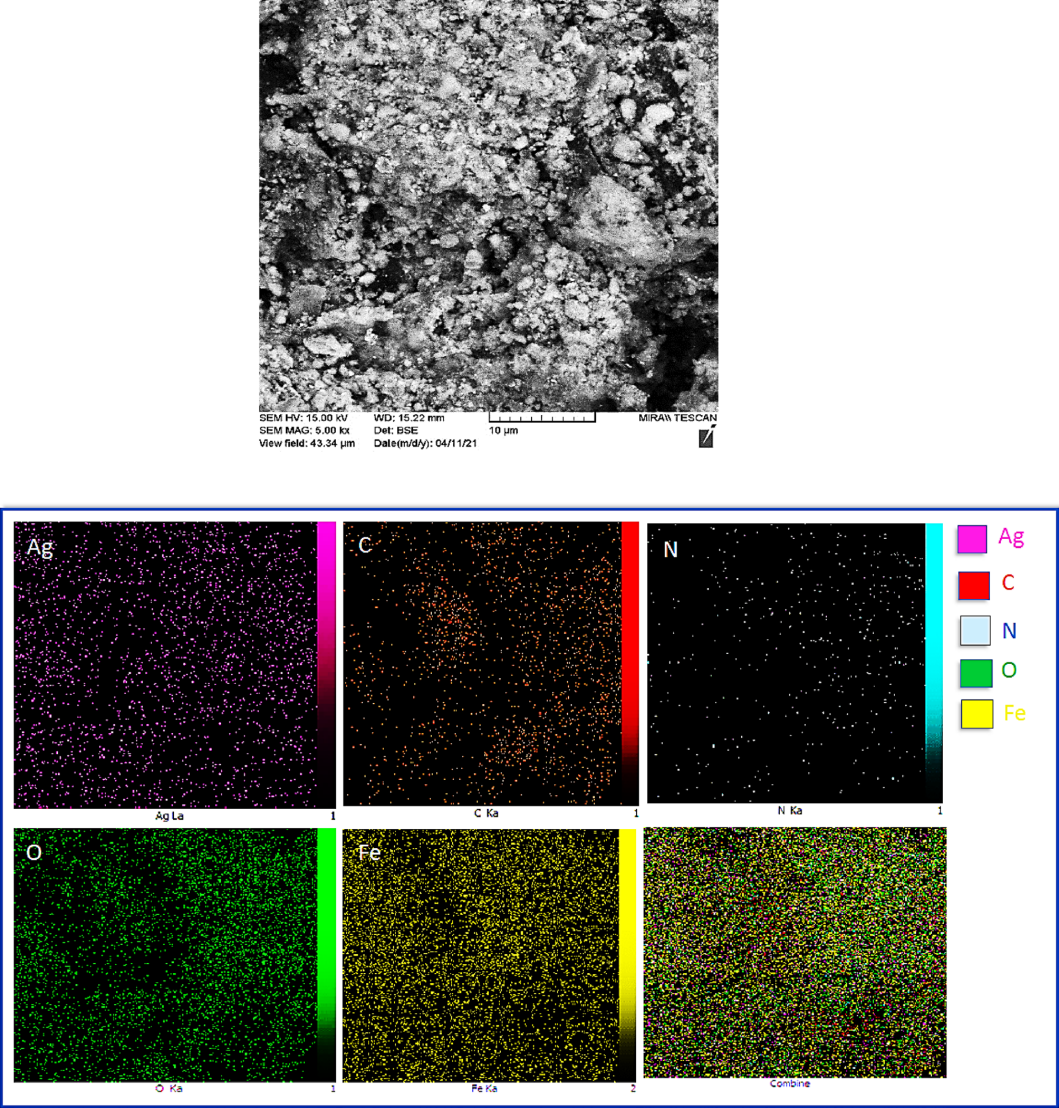
Elemental mapping of biochar/Fe_3_O_4_/PVIm/Ag nanocatalyst.

Furthermore, elemental mappings of biochar/Fe_3_O_4_/PVIm/Ag nnanocatalysts were provided to characterize the catalyst. As shown in Fig. 6, Ag atoms were uniformly dispersed in the catalyst. It showed that the distribution of C and Fe atoms was uniform, therefore elements were uniformly distributed in biochar.

**Figure 7:**
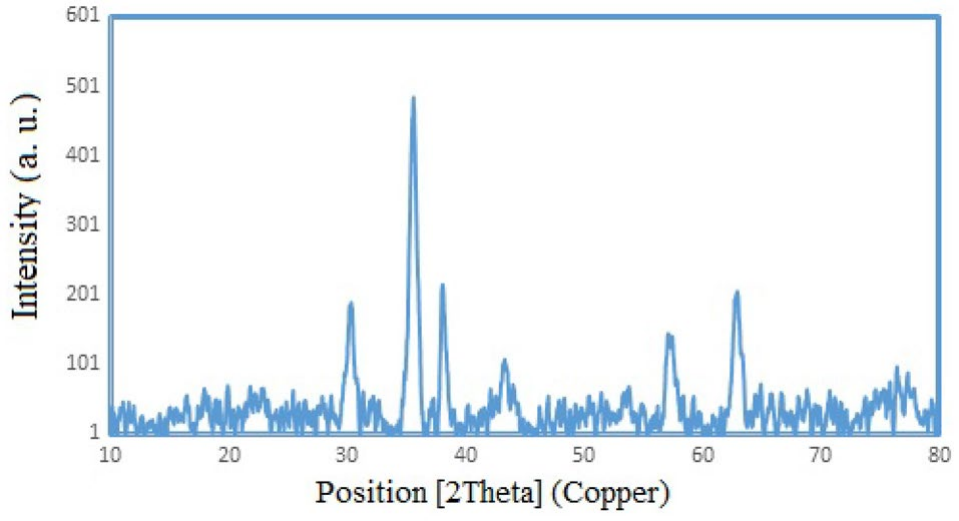
XRD pattern of biochar/Fe_3_O_4_/PVIm/Ag nanocatalyst.

X-ray diffraction (XRD) pattern was performed to approve the crystalline structure of the synthesized biochar/Fe_3_O_4_/PVIm/Ag nanocatalyst (Fig. 7). The biochar displayed a broad peak at 22° (002) related to graphite-like structures (crystalline carbon). The characteristic peaks of Ag nanoparticles were seen at 2θ = 38.28°, 44.40°, 64.57°, and 77.48° corresponding to cube silver lattice in Miller indices i.e. (111), (200), (220), and (311). In addition, characteristic peaks of Fe_3_O_4_ nanoparticles appeared at 2θ = 30.3°, 35.6°, 43.2°, 54.0°, 57.3°, and 63.0° with Miller indices of (220), (311), (400), (422), (511) and (440) respectively. (JCPDS card number 39-1346).

**Figure 8:**
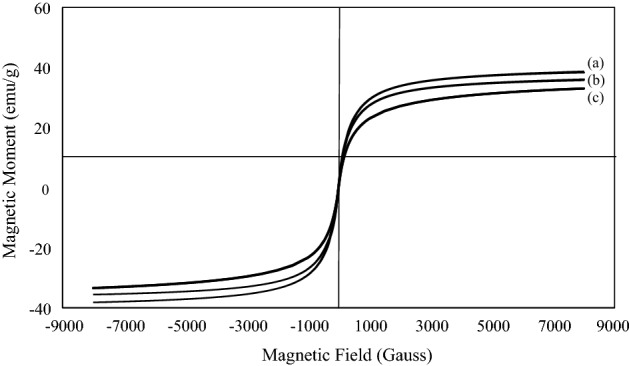
VSM diagram of (**a**) biochar/Fe_3_O_4_, (**b**) biochar/Fe_3_O_4_/PVIm and (**c**) biochar/Fe_3_O_4_/PVIm/Ag.

To assist decide catalyst properties, the magnetic property of biochar/Fe_3_O_4_/PVIm/Ag nanocatalyst, containing a magnetite component was considered by a VSM at ambient temperature (Fig. 8). As illustrated in Fig. 8, the maximum saturation magnetization (Ms) value of biochar/Fe_3_O_4_ was estimated to be 38.7 emu g^−1^ (Fig. 8a) indicating that it has superparamagnetic properties. The Ms was obtained at 38.3 (Fig. 8b) and 33.0 (Fig. 8c) amu g^−1^ for biochar/Fe_3_O_4_/PVIm and biochar/Fe_3_O_4_/PVIm/Ag, respectively, which shows that the saturation magnetization has decreased with the addition of non-magnetic materials. But this reduction is slight, so the nanocatalyst is easily separated by a magnet from the reaction media.

The gotten magnetic hysteresis circle pictorially appears in Fig. 8. It is readily apparent that it is without a hysteresis circle (S-shaped). It is worth noting that the biochar/Fe_3_O_4_/PVIm/Ag can be effortlessly collected by utilizing a magnet, and as a result, the catalyst recovery is facilitated, improving its recyclability.

**Figure 9:**
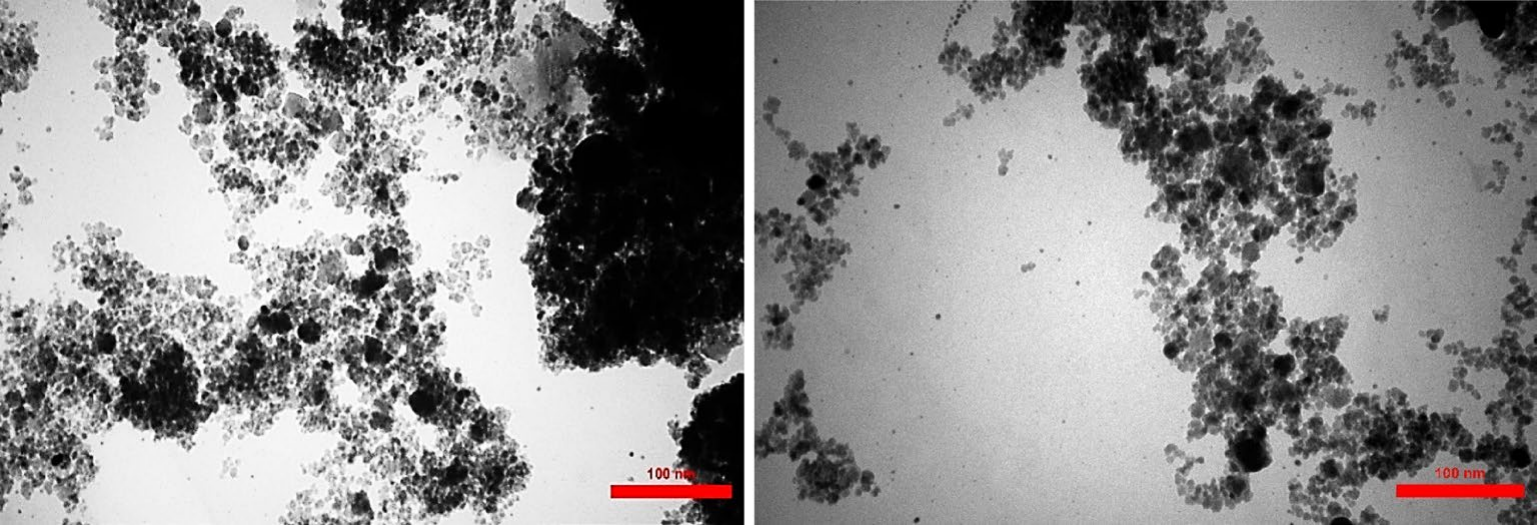
TEM image of biochar/Fe_3_O_4_/PVIm/Ag nanocatalyst.

The TEM image (Fig. 9) of the nanocatalyst, biochar/Fe_3_O_4_/PVIm/Ag, allows the observation of microscopic sheets of biochar. Dark areas on the sheet may indicate the presence of Ag and Fe_3_O_4_ nanoparticles and the formation of biochar/Fe_3_O_4_/PVIm/Ag. In addition, we can confirm that Ag and Fe_3_O_4_ spheres are observed in polyvinyl imidazole and on biochar surface and the immobilization of Ag and Fe_3_O_4_ on biochar and PVIm was successful.

The average diameter of synthesized particles from biochar/Fe_3_O_4_/PVIm/Ag catalyst as proven for each FE-SEM and TEM can be 30–40 nm; moreover, as indicated, the biochar/Fe_3_O_4_/PVIm/Ag confirmed sheet-like morphology.

This result is consistent with the FE-SEM result. Due to the magnetic properties of biochar/Fe_3_O_4_/PVIm/Ag, it is somewhat aggregated which is related to the dispersion of biochar/Fe_3_O_4_/PVIm/Ag as can be seen in the TEM image.

Inductively coupled plasma was used for the examination of Ag content as the main nanocatalyst component. According to the results, the silver content was 0.051%. Comparing the results of ICP and EDS shows the difference between the amount of silver in the catalyst and this is because EDS is a surface and local analysis and gives the amount of silver on the surface, but ICP is a bulk analysis and determines its amount in the whole catalyst.

After the characterization the of synthesized nanocatalyst, which proceeded in eco-friendly chemical processes, we considered the biochar/Fe_3_O_4_/PVIm/Ag as a suitable, easily separable nanocatalyst for a one-pot process of spiro-2-amino-4*H*-pyran (spirochromene) by the multicomponent reaction (Scheme 1).

### Synthesis of spiro-2-amino-4*H*-pryans

The catalytic efficiency of biochar/Fe_3_O_4_/PVIm/Ag was investigated in the formation of ethyl-6-amino-5-cyano-2-methyl-4*H*-pyran-3-carboxylate through the reaction of the following three related components which consists of 1,3-dicarbonyl compounds acting as isatin, malononitrile/ethyl cyanoacetate, 5-chloro isatin, and barbituric acid, dimedone, ethyl acetoacetate, *α*-naphthol, β-naphthol, 4-hydroxyqumarin and three-methyl-1*H*- isatin and its derivative pyrazole-5 (4*H*)-on.

**Scheme 2:**
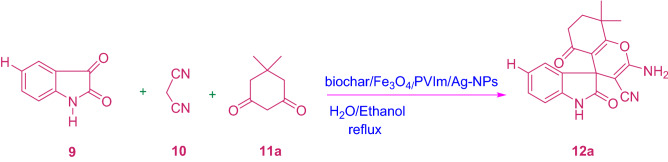
Synthesis of 12a derivative.

To optimize different factors affecting the reaction, different parameters which include catalyst amount, solvent type, and temperature were investigated as a selected reaction including isatin, malononitrile, and dimedone (Table 1, and Scheme 2).

**Scheme 3:**
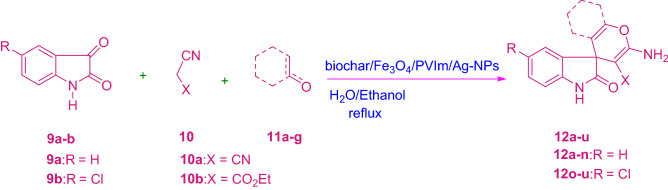
Synthesis of 12a-u derivatives.

**Scheme 4:**
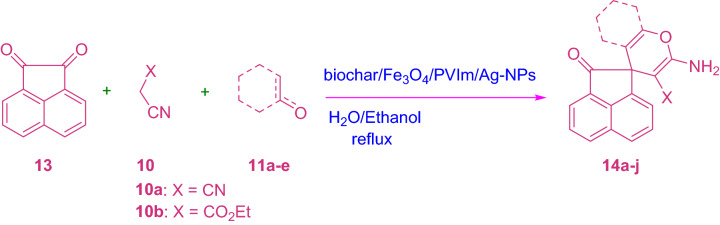
Synthesis of 14a-j derivatives.

To investigate the efficiency and ability of this catalytic MCR, isatin includes isatin and 5-chloroisatin, acenaphthoquinone, malononitrile or ethyl cyanoacetate, and cyclic ketone, as well as barbituric acid, dimethyl ketone, 3-methyl-1*H*-pyrazole-penta (4*H*)-one and tetrahydroxycoumarin, acyclic 1,3-dicarbonyl compound and ethyl acetoacetate, α-naphthol/β-naphthol, 4-hydroxy odor bean, barbituric acid and trimethyl-1H-pyrazole-5 (4*H*)-one, were chosen to synthesize the desired products (Tables 2 and 3, and Schemes 3 and 4).

**Table 3:** Synthesis of 14a-j derivatives in the presence of biochar/Fe_3_O_4_/PVIm/Ag.

Entry	Ar	X	Time (min)	Product	Yield (%)	m.p. (ºC) Obs	m.p. (ºC) Lit
**1**		CN	9	**14a**	98	268–271	268–270^53^
**2**		CN	9	**14b**	95	> 300	> 300^41^
**3**		CN	13	**14c**	94	287–290	288–292^54^
**4**		CN	11	**14d**	96	297–299	298–299^55^
5		CN	30	**14e**	88	> 300	> 300^56^
**6**		CN	22	**14f.**	86	> 300	> 300^28^
7		CO_2_Et	18	**14g**	92	> 300	> 300^53^
**8**		CO_2_Et	27	**14h**	95	260–263	259–262^53^
**9**		CO_2_Et	32	**14i**	86	240–243	240–242^57^
**10**		CO_2_Et	25	**14j**	85	246–248	247–248^53^

First, the reaction of isatin and malononitrile with 3-methyl-1*H*-pyrazole-5(4*H*)-one/4-hydroxycoumarin or barbituric acid/dimedone/ethyl acetoacetate or α-naphthol/β-naphthol was investigated. In the process it did bold out in that Table 2, this MCR went smoothly and produced the desired compound (12a-u) in high yields, with very short response times.

These reactions were catalyzed by biochar/Fe_3_O_4_/PVIm/Ag, isatin was reacted to ethyl cyanoacetate and barbituric acid/dimedone/ethyl acetoacetate or 4-hydroxy Reacted with coumarin/3-methyl-1*H*-pyrazole-5(4*H*) -one or α-naphthol/β-naphthol.

It appears that the predicted products (12a-u) were obtained in high yields. In addition, it was investigated whether other isatin derivatives specifically 4-chlorination are effective. The detail of the three-component reaction of isatin and ethyl cyanoacetate/malononitrile using barbituric acid/dimedone/ethyl acetoacetate or 3-methyl-1*H*-pyrazole-5 (4*H*)-one/4-hydroxycoumarin or α-naphthol/β-naphthol can be seen in Table 2 (10a-u).

The required factors were given in high yield, regardless of the influence of the properties of the substituents of isatin. The less spiro-4*H*-pyran (12a-j) was produced when acetyl naphthoquinone (13) was used (Table 3). It is recognizable that the reaction with ethyl cyanoacetate enforced an extended response time than the reaction via malononitrile, which is probably due to its low reactivity (Table 3).

The method illustrates to a normal successive reaction wherein the isatin (9), first, connected to malononitrile (10) combines with isatylidene malononitrile in the presence of biochar/Fe_3_O_4_/PVIm/Ag in EtOH/water. This step was named fast Knoevenagel condensation.

**Scheme 5:**
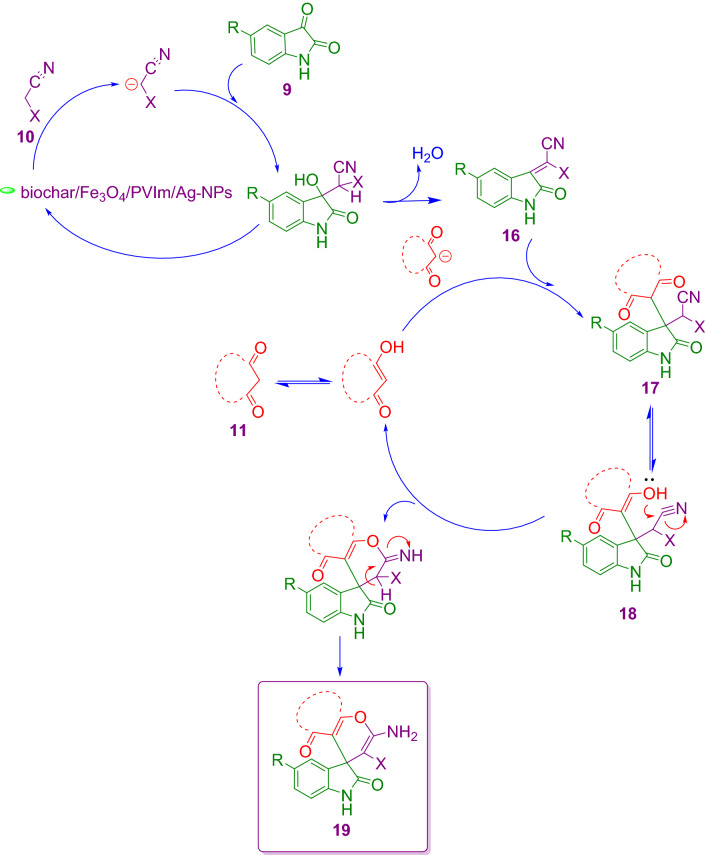
Proposed component for the blend of spiro subsidiaries **19**.

Intermediate (17) was formed via a Michael addition mechanism by adding compound (11) to compound (16). Then the hydroxyl group attacked the cyanide group through an intermolecular reaction in compound 18 and finally after tautomerization the final product (19) was formed. Of course, the reaction may be a cascading reaction through Knoevenagel Condensation/Michael Addition. Scheme 5 proposed component for the blend of spiro subsidiaries 19. Scheme 5 proposed component for the blend of spiro subsidiaries 19. Scheme 5 proposed component for the blend of spiro subsidiaries 19.

**Scheme 6:**
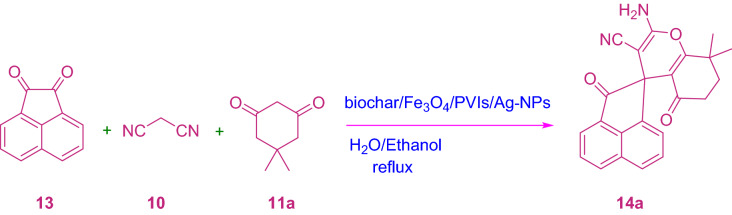
Synthesis of 14a derivative.

To study the efficiency of the synthesized nanocatalyst, its catalytic performance was compared with the others mentioned in Table 4 (also Scheme 6). The catalytic strength of our specific catalyst (biochar/Fe_3_O_4_/PVIm/Ag) is compared with the recently detailed MCRs including acenaphthoquinone, malononitrile, and dimethyl ketone to use 2′-amino-tetrahydro-2*H*-spiro[acenaphthylen-1,4′-chromeno]-3′-carbonitrile (**14a**) and different catalysts, such as CaCl_2_^57^, Fe_3_O_4_@Cs-CsO_3_H^56^_,_ Na_2_EDTA^58^, HAuCl_4_^.^3H_2_O^59^, Meglumine^41^, Fe_2_O_3_^60^, HEAA^61^, Cu(OAc)_2_.H_2_O^46^, Fe_3_O_4_@CS-SO_3_H NPs^62^, Amb-400Cl (IRA-400 Cl)^63^, C_4_(DABCO-SO_3_H)_2_.4Cl^64^, Carbon-SO_3_H^65^, 1-butyl-3-methylimidazolium hydroxide ([bmim][OH]^66^, DBU^67^, (SB-DBU)Cl^68^, PEG-Ni nanoparticles^69^, trisodium citrate dihydrate^70^, PC/AgNPs^35^ and new catalyst biochar/Fe_3_O_4_/PVIm/Ag. It seems that our new catalyst is better than other catalysts in more items.

**Table 4:** Synthesis of 2′-amino-8′,8′-dimethyl-2,5′-dioxo-5′,6′,7′,8′-tetrahydro-2*H*-spiro[acenaphthylene-1,4′-chromene]-3′-carbonitrile (**14a**) in the presence of biochar/Fe_3_O_4_/PVIm/Ag.

Entry	Catalyst	Catalyst amount (g)	Time (min)	Temperature	Solvent	Yield (%)	Lit.^ref^
1	CaCl_2_	0.02	50	r.t.	Ultrasonic	96	^57^
2	Fe_3_O_4_@Cs-SO_3_H	0.02	300	Reflux	H_2_O/EtOH	98	^56^
3	Na_2_EDTA	0.01	10	70 °C	Solvent-free	94	^58^
4	HAuCl_4_^.^3H_2_O	0.05	30	70 °C	PEG 400	96	^59^
5	Meglumine	0.05	20	r.t.	H_2_O/EtOH	93	^41^
6	Fe_2_O_3_	0.02	240	90 °C	Solvent-free	84	^60^
7	HEAA	0.02	60	90 °C	H_2_O	92	^61^
8	Cu(OAc)_2_.H_2_O	0.02	300	80 °C	Solvent-free	84	^46^
9	Amb-400Cl (IRA-400 Cl)	0.02	10	Reflux	H_2_O	95	^63^
10	Fe_3_O_4_@CS-SO_3_H NPs	0.02	5	r.t.	H_2_O/EtOH	92	^62^
11	C_4_(DABCO-SO_3_H)_2_.4Cl	0.01	12	90 °C	H_2_O	98	^64^
12	1-Butyl-3-methylimidazolium hydroxide ([bmim][OH]		20	r.t.	Solvent-free	92	^65^
13	Carbon–SO_3_H	0.01	180	Reflux	EtOH	91	^66^
14	DBU	0.01	15	Reflux	H_2_O	88	^67^
15	(SB-DBU)Cl	0.05	60	r.t.	EtOH	98	^68^
16	PEG-Ni nanoparticle	0.0235	10	r.t.	PEG	93	^69^
17	Trisodium citrate dihydrate	0.01	300	r.t.	H_2_O/EtOH	92	^70^
18	PC/AgNPs	0.025	10	reflux	H_2_O/EtOH	94	^35^
19	Biochar/Fe_3_O_4_/PVIm/Ag	0.03	8	reflux	H_2_O/EtOH	93	Our work

Moreover, our strategy achieved the required items in a way well yielded and fast response times. In terms of green chemistry, the reusability of this nanocatalyst, in conjunction with utilizing H_2_O/EtOH as an almost green solvent, enables this environmentally friendly and harmless catalyst to be used in mechanical systems.

The relevant synthesis in this research has already been reported in the literature. All steps have been carried out according to organic synthesis principles, such as Michael addition, water removal, and tautomerization^71,72^.

### Reusability of catalyst

**Figure 10:**
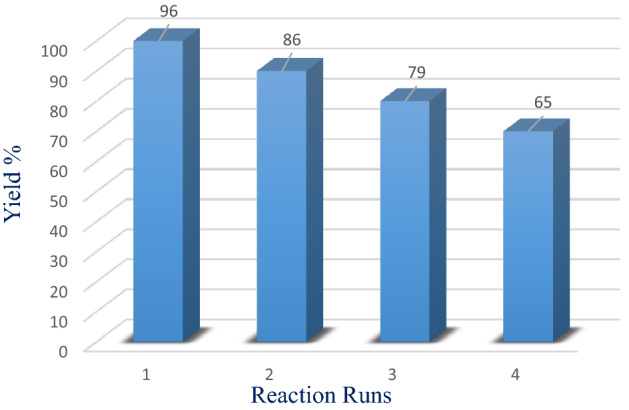
Normal reusability of our new catalyst within the alertness of admixture 10a.

**Scheme 7:**
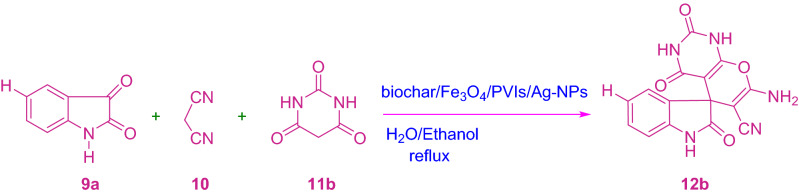
Synthesis of 12b derivative.

We also investigated the importance of the catalyst and its potential for reuse. The reaction between isatin, barbituric acid and malononitrile under optimized conditions was selected to investigate the reusability of the synthesized nanocatalyst. After the end of the reaction, the catalyst was isolated thru an external magnet and washed with ethanol. The separated nanocatalyst was reused inside the next cycle in the similar reaction environment. In this study was observed that the nanocatalyst can be recovered and reutilized in at least 4. In the fourth cycle, the efficiency of the catalyst decreased (Fig. 10, and Scheme 7).

To study the stability of the nanocatalyst in the optimal reaction conditions, ICP was taken and the amount of silver decreased from the initial value of 0.051–0.042%. This result shows that the catalyst has relatively acceptable stability.

## Conclusion

In conclusion, the above-presented investigation made accessible an efficient and quick approach for the synthesis of Ag nanoparticles immobilized onto the magnetic biochar/polyvinyl imidazole to get an eco-friendly nanocomposite with a good activity, green, and heterogeneous catalyst. The possibility of obtaining and compositing three materials, biochar, polymer, and nanoparticles, has the ability to access a green compound in the imminent need for the design of efficient and environmentally friendly catalysts. Biochar/Fe_3_O_4_/PVIm/Ag as a new catalyst was effectively utilized as a nanocatalyst within the production of spirochromenes. This catalyst was used to obtain the desired products with high yield. The biochar/Fe_3_O_4_/PVIm/Ag nanocatalyst was separated easily by an external magnet. The catalyst was recoverable and reusable for 4 runs without a significant reduction in effectiveness. Another advantage of this catalytic system had been that it could be carried out under gentle reaction conditions in very brief times, in conjunction with a simple process, and the good stability under the optimized conditions. In summary, as the technical advantages of nanotechnology rapidly change from laboratory to large-scale industrial development, nanomaterials are used in all synthesis applications.

## Data Availability

All data generated or analyzed during this study are included in this published article.
